# An Audit on Telephone Referrals to Beechcroft, a Step 5 Regional Child and Adolescent Mental Health Inpatient Unit

**DOI:** 10.1192/bjo.2024.545

**Published:** 2024-08-01

**Authors:** Andrew Breakwell, Claire Kelly, Lucy Brakspear, Owen McMurray, Karolina Szczygiel

**Affiliations:** Beechcroft, Belfast, United Kingdom

## Abstract

**Aims:**

To audit telephone referrals to Beechcroft inpatient unit.

Beechcroft inpatient unit is a step 5 regional child and adolescent mental health inpatient unit in Belfast. It receives a large volume of referrals from across all five health and social care trusts in Northern Ireland. The process of referral to Beechcroft can vary between trusts and clinicians; the majority of admissions are emergency. The demand for beds has risen by 30% since 2019. Emergency admissions are commonly telephone referrals whilst others submit written referrals. The referrals process is managed by the ward sisters, as there is no bed manager post. Referrals are discussed with a consultant psychiatrist.

Referrals received often lack key clinical information, which makes decisions around appropriateness of admission or prioritising multiple referrals difficult. Furthermore, as the admitting doctor relies on this information, missing clinical information could result in patient safety issues.

**Methods:**

24 telephone referrals were recorded between August to December 2023. 5 referrals were excluded for either no request for a bed (3) or telephone update following previous written referral (2). 19 telephone referrals were analysed across 7 different criteria as below, based on necessary information.

Criteria 1 Patient identifiable information

Criteria 2 Source of referral/referrer details

Criteria 3 Current location of patient

Criteria 4 Legal status

Criteria 5 Presenting symptoms

Criteria 6 Working diagnosis

Criteria 7 Risks warranting admission

**Results:**

Yes    No   %Yes

Criteria 1   19    0   100

Criteria 2    19    0   100

Criteria 3   13    6   68

Criteria 4    14   5   74

Criteria 5   18   1    95

Criteria 6    2   17   11

Criteria 7   15   4   79

**Total**     **100   33   75.2**

Patient identifiable information and source was documented in all referrals. Only 10% of referrals included a working diagnosis. Location of patient, legal status and risks warranting admission were documented between 68 and 78%.

**Conclusion:**

Crucial information such as working diagnosis was missing in 90%. Risks or legal status missing in up to a quarter of referrals. This has an impact on timely access, bed flow and potentially patient safety.

A need for improvement in receiving and documenting telephone referrals has been identified. To aid improvement in patient safety and flow, a bed manager for in hours has now been appointed. A standardised proforma for recording data will be developed by inpatient staff in collaboration with community staff to include the above criteria. A re-audit will be carried out following these service improvements.

